# Analyzing Sustainable 3D Printing Processes: Mechanical, Thermal, and Crystallographic Insights

**DOI:** 10.3390/polym16101364

**Published:** 2024-05-10

**Authors:** Alexandra-Ileana Portoacă, Alin Diniță, Maria Tănase, Alexandru Săvulescu, Elena-Emilia Sirbu, Catălina Călin, Gheorghe Brănoiu

**Affiliations:** 1 Mechanical Engineering Department, Petroleum-Gas University of Ploiești, 100680 Ploiesti, Romania; alexandra.portoaca@upg-ploiesti.ro; 2 Automation, Computers and Electronics Department, Petroleum-Gas University of Ploiești, 100680 Ploiesti, Romania; asavulescu@upg-ploiesti.ro; 3 Chemistry Department, Petroleum-Gas University of Ploiești, 100680 Ploiesti, Romania; elena.oprescu@upg-ploiesti.ro (E.-E.S.); catalina.calin20@yahoo.com (C.C.); 4 Petroleum Geology and Reservoir Engineering Department, Petroleum-Gas University of Ploiești, 100680 Ploiesti, Romania; gheorghe.branoiu@upg-ploiesti.ro

**Keywords:** DSC, TGA, XRD, energy consumption, crystallinity, mechanical characteristics, design of experiments, colored thermoplastic aliphatic polyester

## Abstract

In this study, the objective was to optimize energy consumption in the fused deposition modeling (FDM) 3D printing process via a detailed analysis of printing parameters. By utilizing thermal analysis techniques, this research aimed to identify lower printing temperatures that could lead to reduced energy usage. Experimental analysis was conducted using a three-level L9 Taguchi orthogonal array, which involved a systematic combination of different extruder temperatures and cooling fan capacities. Furthermore, the research incorporated differential scanning calorimetry (DSC) and X-ray diffraction (XRD) methods to analyze the thermal properties and crystallinity of the 3D-printed specimens. The results indicated that temperature was a key factor affecting crystallinity, with samples printed at 190 °C and 60% fan capacity showing the highest mean values. By conducting a multi-objective desirability analysis, the optimal conditions for maximizing ultimate tensile strength (UTS), tensile modulus, and elongation at break while minimizing energy consumption for PLA 3D-printed samples were determined to be a temperature of 180 °C and a fan speed of 80%.

## 1. Introduction

Additive manufacturing (AM) encompasses a group of processes that facilitate the incremental production of objects based on three-dimensional (3D) model data, layer by layer. This is in contrast to subtractive manufacturing, the predominant method in most manufacturing operations. An advantage of AM lies in its ability to create functional components with complex geometries that prove challenging to produce using traditional methods. Fused deposition modeling (FDM), among various AM techniques, stands out as a highly popular and extensively utilized method for producing parts using plastic materials [1,2,3]. Various factors within the FDM process can impact the mechanical properties of 3D-printed parts. For instance, parameters such as layer thickness [4,5,6], infill density, printing temperature, and filament type [7,8,9,10,11,12,13] all play a significant role in determining the mechanical characteristics of the produced object. Also, the post-processing treatments can considerably influence the mechanical behavior of 3D-printed parts [14,15,16,17]. On the other hand, the sustainability and energy efficiency of AM have become increasingly critical considerations in contemporary industrial practices. Numerous scientific research [5,6,18,19,20,21,22,23,24] has concentrated on this aspect of AM technology, aiming to establish a compromise between energy efficiency and mechanical strength.

Shifting focus back to the correlation between production costs and the fabrication of printed components, the current emphasis, as highlighted in reference [6], is on optimizing energy efficiency and cost reduction within the manufacturing process, particularly in the domain of material extrusion additive manufacturing. This optimization is essential for achieving both sustainability and cost-effectiveness in production. Notably, MEX (material extrusion) 3D printing consistently demonstrates its capability to produce high-quality parts, especially when utilizing expensive high-performance polymers with applications in the biomedical, automotive, and aerospace sectors. The referenced scientific paper explores the effect of three crucial parameters (layer thickness, nozzle temperature, and printing speed) on energy consumption (directly influencing production costs) and tensile strength of 3D-printed PEEK (Polyetheretherketone) parts. The dominant parameter influencing the tensile strength and energy printing consumption was found to be layer thickness.

The research outlined in [18] extensively investigates the impact of seven universal and machine-agnostic 3D printing configurations on both the energy usage and mechanical properties of parts manufactured from PLA (polylactic acid)using the MEX 3D printing method. The results underscored that printing speed and layer thickness had the most substantial impact on energy consumption in the study. Additionally, infill density and orientation angle were identified as the primary factors influencing compressive strength.

Furthermore, in a related study [19], statistical modeling tools were employed to evaluate various metrics associated with compression and energy consumption in 3D printing. These metrics encompassed printing time, weight, energy printing consumption, specific printing energy, specific printing power, compression strength, compression modulus of elasticity, and toughness. Among the analyzed factors, layer thickness emerged as the most influential control parameter, while nozzle temperature and raster deposition angle exhibited lesser impact on the outcomes.

Study [20] quantifies energy consumption for producing 3D-printed PLA parts, providing valuable insights for assessing the process’s sustainability. Six machine-independent parameters with three levels were analyzed, revealing a significant impact on energy consumption, with differences of up to 250% among the studied cases. The study also evaluated the effect of these parameters on the flexural strength of PLA parts, finding substantial variations of up to 300%. Although efforts were made to identify optimal 3D-printing settings for balanced performance, achieving both minimal energy consumption and high flexural strength proved challenging. High layer thickness values were associated with minimum energy consumption, although they produced parts with average flexural strength.

Vidakis et al. [21] investigated the impact of six control factors (infill density, raster deposition angle, nozzle temperature, print speed, layer thickness, and bed temperature) on the energy performance and mechanical properties of Poly[methyl methacrylate] in 3D printing. The study found that raster deposition angle and printing speed were the most influential parameters for tensile strength, while layer thickness and printing speed significantly affected energy consumption. Quadratic regression models were developed for each response metric, allowing the identification of an optimal balance between energy efficiency and mechanical strength in engineering applications. Similarly, article [22] addresses the contemporary industrial demand for sustainability and energy efficiency in AM, particularly focusing on the need for 3D-printed parts with strong mechanical properties, especially in high-performance polymers like polycarbonates used in MEX. The study investigates the impact of seven control parameters on the energy consumption and compressive performance of polycarbonate in MEX AM. Using a three-level L27 Taguchi experimental design with 135 experiments, the research identifies layer thickness and infill density as the most influential factors in energy consumption, while infill density and orientation angle significantly affect compressive strength.

PLA filament is widely favored in the 3D printing market due to its affordability, smooth printing operation, and satisfactory physical properties [25]. It is crucial to carefully design and control the PLA crystallization temperature as an essential parameter. Throughout the processing conditions, changes in the PLA crystallization temperature can influence the formation of specific crystalline structures in PLA, favoring one structure over another [26].

The correlation between crystallinity and mechanical performance in 3D-printed materials is a critical aspect of understanding how the structural arrangement of a material at the molecular level influences its mechanical properties. The degree of crystallinity in a 3D-printed object can significantly impact its strength, stiffness, and other mechanical characteristics.

In [27], a positive correlation was observed between crystallinity data and impact strength data, indicating that higher crystallinity resulted in enhanced impact strength.

Ansari et al. [25] have found that crystallinity has an inverse relationship with print speed, with higher crystallinity achieved at lower print speeds. Additionally, infill density plays a significant role in influencing crystallinity. A lower infill density of 50% is associated with higher crystallinity, but this decreases significantly when increasing the infill density to 75%. Notably, both the grid and trihexagonal patterns exhibit higher crystallinity, while the triangular pattern is linked to the lowest crystallinity. Magri et al. [1] conducted research on the mechanical properties of carbon fiber-reinforced PLA composites. They found that the inclusion of fiber reinforcement led to enhanced tensile properties. The maximum tensile strength was achieved at the highest nozzle temperature (230 °C). Additionally, their findings indicated that annealing increased crystallinity, and a lower cooling rate resulted in a higher crystalline structure in the polymer.

The literature extensively underscores that the quality and performance of 3D-printed parts are inherently tied to the intricacies of chosen process parameters. The nuanced interplay of variables such as layer thickness, printing speed, and infill density plays a pivotal role in shaping the final outcome. This realization is of paramount importance in the landscape of AM, where precision and efficiency are sought to be optimized continually. Recognizing the multifaceted impact of these parameters on mechanical properties and energy consumption, the literature emphasizes the ongoing need for systematic investigation and optimization, particularly in the realm of AM.

In the context of this imperative, the present work assumes significant relevance. The primary objective was to derive optimized printing parameters that not only ensure comparable mechanical performance but also contribute to a reduction in energy consumption. This dual focus on performance and energy efficiency is vital for leading additive manufacturing towards a sustainable and cost-effective trajectory. As industry increasingly prioritizes environmental responsibility, achieving this delicate balance becomes imperative for the widespread adoption and continued growth of 3D printing technologies.

Moreover, this exploration delves into the realm of material science via the application of differential scanning calorimetry (DSC) and X-ray diffraction (XRD). By utilizing DSC to evaluate the crystallography of both colored thermoplastic aliphatic polyester filament and 3D-printed specimens, the study extends beyond conventional analyses. Establishing a direct correlation between crystallinity and mechanical properties offers a profound understanding of how the molecular structure influences the final product’s strength, stiffness, and other mechanical characteristics. This linkage between crystallinity and performance is a critical aspect in adjusting printing parameters to control the crystalline structure of the material.

The use of DSC and XRD provides nuanced insights that extend beyond traditional mechanical testing. The targeted adjustments informed by crystallinity data offer a pathway to enhance not only the mechanical performance of printed objects but also their sustainability. This dual optimization aligns with the evolving expectations of industries seeking to embrace 3D printing as a versatile and environmentally responsible manufacturing method.

By emphasizing the crucial relationship between process parameters, mechanical performance, and energy consumption, the present study contributes valuable insights into the global perspective of sustainable manufacturing. The integration of advanced analytical techniques, such as DSC and XRD, improves the understanding of material behavior, allowing for more precise adjustments and, consequently, promoting the evolution of additive manufacturing towards higher performance and heightened sustainability.

## 2. Materials and Methods

**Figure 1:**
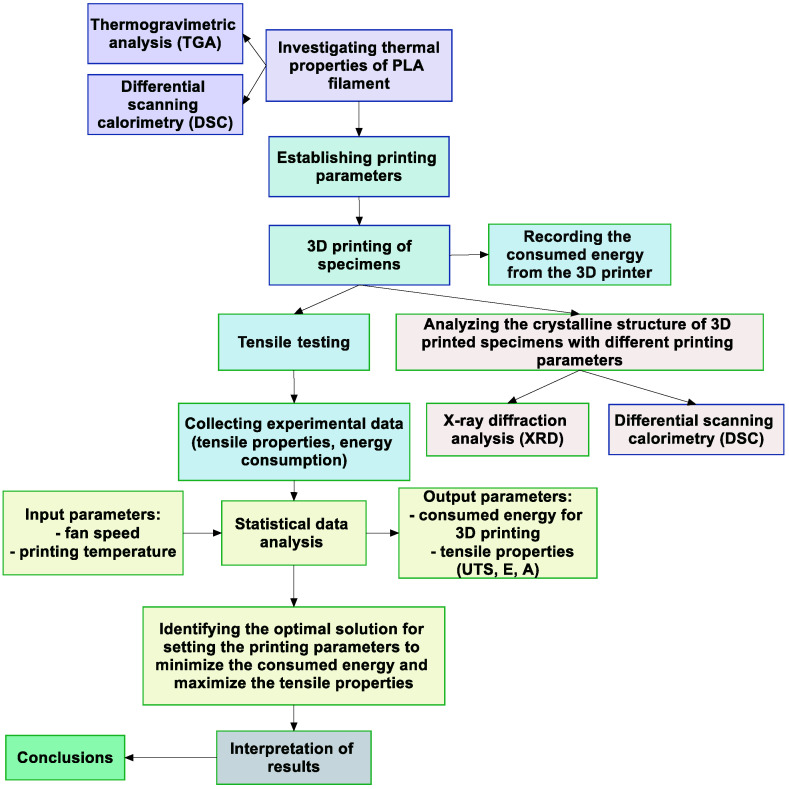
Flow chart diagram illustrating the stages of the performed investigation.

As mentioned above, the aim of the present work was to identify the influence of printing parameters (extrusion temperature and fan speed) on the mechanical behavior and energy consumption of 3D-printed colored thermoplastic aliphatic polyester objects, so as to establish the optimal solution using the steps presented in Figure 1.

The performed investigation begins with an analysis of the thermal properties of PLA filament (using the characteristics given in Table 1) via Thermogravimetric Analysis (TGA) and differential scanning calorimetry (DGA). The specific used filament was Polymaker PolyTerra Polymaker (Utrecht, The Netherlands) with 1.75 mm diameter.

The next step involves establishing the printing parameters, followed by the fabrication of tensile test specimens. These specimens are then subjected to tensile testing, and the experimental data (tensile properties and energy consumption) are collected. Using these data, a statistical analysis is performed, focusing on input parameters such as fan speed and printing temperature, with the aim of identifying the optimal settings for these parameters that minimize energy consumption while maximizing tensile properties. Finally, the conclusions are drawn from the interpretation of the results, which should ideally provide the optimal solution for the 3D printing parameters that balance energy efficiency with material performance.

### 2.1. DSC Analysis

In order to ensure the stability of the colored thermoplastic aliphatic polyester polymer and prevent any degradation that could potentially influence the experimental results, the temperatures used in the extrusion processes were evaluated for their impact on thermal properties. The crystallization behavior of the material was analyzed with a DSC 3+ Star system from METTLER TOLEDO (Leicester, England) under a N_2_ atmosphere between 10 and 260 °C with 10 °C/min.

The investigation into the melting and crystallization characteristics of the investigated material involved analyzing the endothermic and exothermic peaks in the DSC thermograms. The degree of crystallinity (*X_c_*) was determined based on the information derived from the DSC thermogram using equation [25]:

(1) $$X_{C}=\frac{\Delta H_{m}}{w\cdot \Delta H_{m}^{100\%}}\cdot 100\left[\%\right]$$

where Δ*H_m_* is the measured melting enthalpy of PLA specimen [J/g], Δ*H_m_*^100%^ is the melting enthalpy for 100% crystalline PLA (93.7 J/g [25]), and *w* is the mass fraction of PLA in the analyzed sample.

### 2.2. 3D Printing Process

The printing process utilized the Raise E2 3D printer, characterized by a volume capacity of 330 × 240 × 240 mm. The particular printing parameters employed in this study are detailed in Table 2, with the build orientation set as X–Y model lines oriented at a 45° angle.

**Figure 2:**
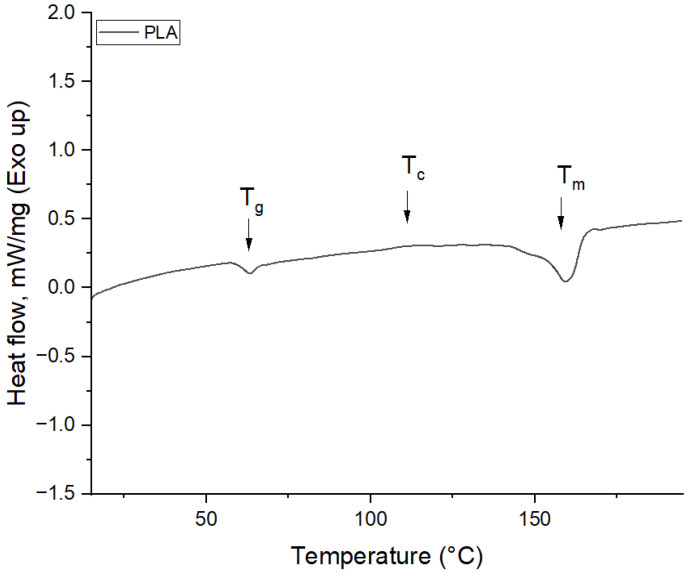
DSC analysis on the colored thermoplastic aliphatic polyester filament (first heating scan).

Extruder temperature and fan speed were variable parameters. The extrusion process parameters for the samples were precisely defined by setting the extruder temperatures at 160 °C, 170 °C, and 180 °C. These values for extruder temperatures were chosen based on the thermodynamic analysis of the filament (given in Supplementary Material Figure S1: TGA curve for filament), which presented the glass transition temperature (*T_g_*) at 62.85 °C, cold crystallization temperature (*T_c_*) at 112.40 °C and melting temperature (*T_m_*) at 160.34 °C (Figure 2). Therefore, the authors chose the range of extrusion temperature between the melting temperature obtained from DSC analysis (160.34 °C) and the extrusion temperature indicated by the filaments provider (210 °C), presented in Table 1.

The selected temperatures are intentionally maintained below 210 °C to achieve a dual objective: firstly, to ensure the thermal integrity of the filament during the extrusion process, and secondly, to enhance the energy efficiency of the system by minimizing the power consumption required for extrusion.

**Figure 3:**
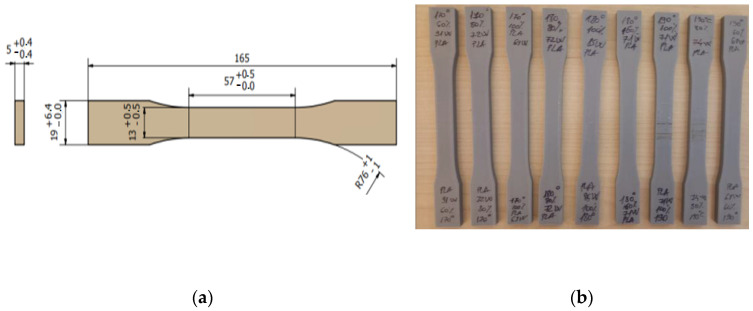
The 3D-printed specimens: (**a**) shape and dimensions of specimens; (**b**) specimens used for testing.

The geometrical characteristics and dimensions of the specimen are shown in Figure 3a, and the printed specimens are presented in Figure 3b.

### 2.3. Energy Consumption Measurement

**Figure 4:**
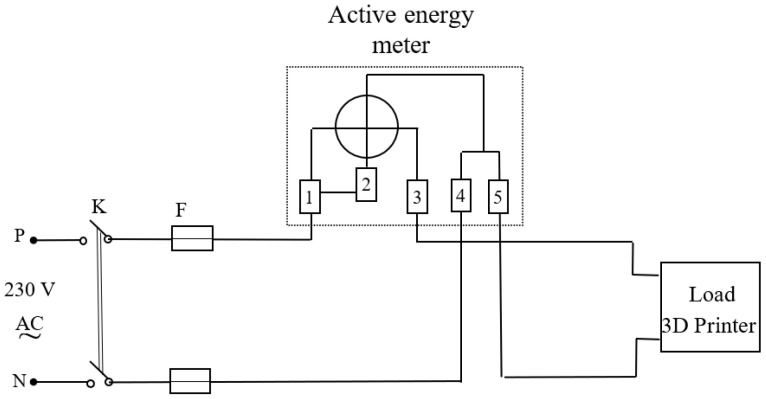
Wiring diagram for measuring the active energy consumed by the 3D printer.

Figure 4 shows the electrical diagram used to measure the active energy consumed by the 3D printer.

**Figure 5:**
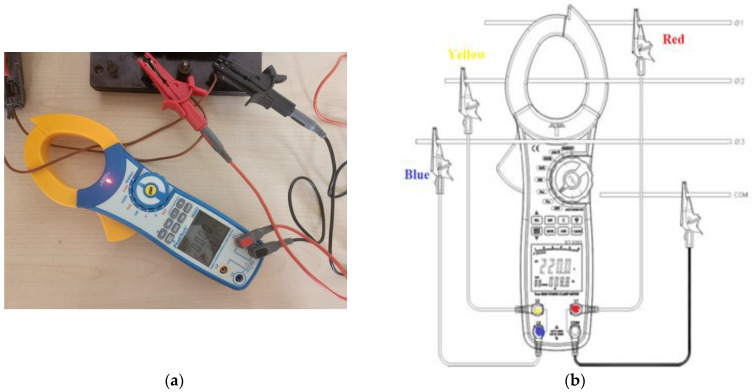
Three-phase digital power clampmeter PeakTech P1660: (**a**) aspect; (**b**) installation in the 3-phase circuit.

The equipment used in the scheme from Figure 4 are as follows:

- K—voltage disconnect switch;
- F 10A—fuse;
- Active energy meter—it uses a 3-phase digital power clampmeter PeakTech P1660 (Distrelec, Vienna, Austria) (with active energy measurement function), mounted in a single phase (see Figure 5).

For connecting the power clampmeter P1660 in a single-phase circuit in order to measure the active energy, only two voltage test leads are connected, one on the phase on which the current is measured (named Yellow in Figure 5) and the other on the neutral conductor.

The used power clampmeter PeakTech P1660 measures AC voltage *V*, frequency *f*, current *I* on the phase conductor surrounded by amperometric clamp, active power *P*, reactive power *Q* and apparent power *S*, power factor *cosφ*, as well as the active energy *W_a_* consumed by the load.

Some specifications of power clampmeter P1660 are:

✓ Measurement rate 2× per sec.;
✓ Voltage range: 100 V, 400 V, 750 V with voltage resolution 0.1 V;
✓ Current range: 40 A, 100 A, 400 A and current resolution 0.1 A;
✓ Resolution 0.001 kWh when measuring energy.

By definition, active energy is the integral of active power, calculated for a time interval [*t*_1_, *t*_2_]:

(2) $$W_{a}=\int_{t_{1}}^{t_{2}}Pdt$$

and the active power *P* is

(3) P=VIcosφ

where *V* and *I* are root-mean-square (RMS) values of voltage and current intensity, and *cosφ* is the power factor.

The voltage and current values measured by the clampmeter, which are also included in the calculation of power and energy, are true RMS values, which indicates better measurement accuracy, considering that voltages and currents are not always perfectly sinusoidal.

### 2.4. XRD Analysis

In order to study the structure of the analyzed samples, a D8 Advance diffractometer (Bruker-AXS, Karlsruhe, Germany) with Cu-Kα radiation (λ = 1.54 Å) was used. The tests were performed on fragments from the 3D-printed specimens in the measurement range (2θ) 10–70°. The equipment with θ-θ configuration and Bragg–Brentano geometry was operated at 40 KV and 40 mA, with scanning conditions: step 0.1° and scan speed 0.1°/5 s. The measurements were carried out via the XRD Commander, and the raw files were obtained. DIFFRAC.EVA v14 software and PDF-ICDD database were used for the qualitative interpretations, and the Rietveld refinements (quantitative interpretations) were run in the TOPAS 4.1 software. Although the X-ray spectra show the same trend, a difference can be seen depending on the temperature and the cooling rate.

The degree of crystallinity *X_C_* in XRD spectra was calculated using the equation

(4) $$X_{c}=\frac{\sum A_{crys}}{\sum A_{crys}+A_{amorph}}$$

where *A_crys_* is the fitted areas of the crystal phase, and *A_amorph_* is the fitted areas of the amorphous phase [28].

### 2.5. Tensile Testing

**Figure 6:**
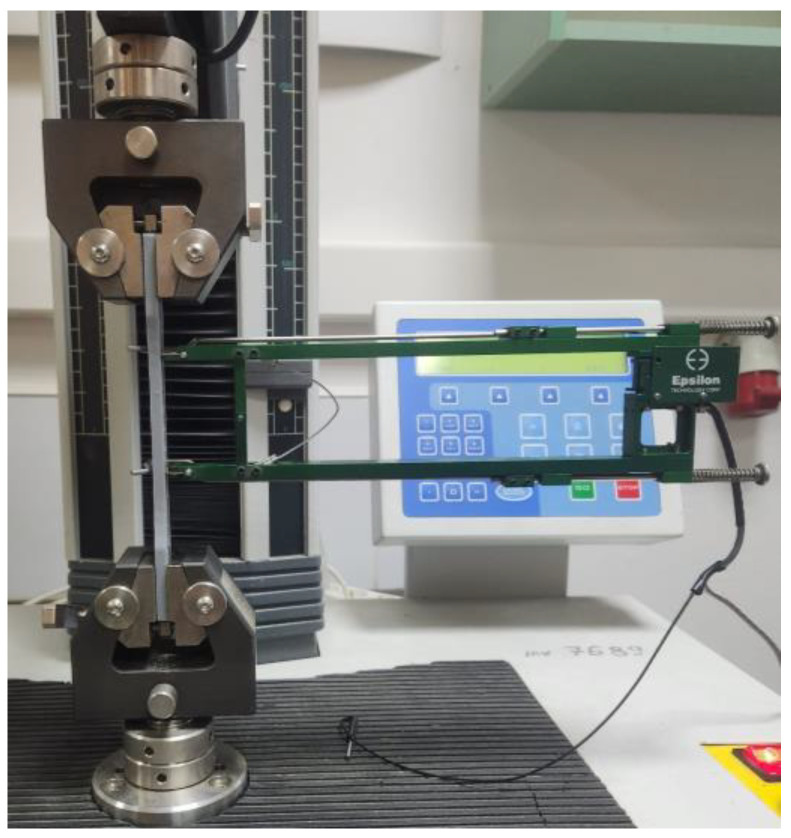
Tensile experiment.

The 9 groups of specimens, each containing 3 samples (printed with 0.2 mm layer thickness, 50% infill percentage, and 2 shells), underwent mechanical testing to establish the main material characteristics, including ultimate tensile strength (*UTS*), elongation at break (*A*), and tensile Young’s modulus (*E*). Tensile tests were conducted using an electro-mechanical machine equipped with a 2.5 kN force cell, operating at a speed of 5 mm/min, and the elongation at break was measured with an axial extensometer (see Figure 6).

### 2.6. Design of Experiments and Optimization

To optimize the 3D printing process and enhance the tensile properties of printed materials while minimizing energy consumption, a systematic approach utilizing the Design of Experiments (DOE) was employed. The primary objective was to maximize tensile properties (UTS, ultimate tensile strength; E, Young’s modulus; and A, elongation at break), encompassing strength and flexibility while concurrently minimizing the energy consumed during the printing process. The factors under investigation were extrusion temperature and fan speed, recognized as pivotal parameters influencing the printing outcome. A full factorial experimental design was chosen to comprehensively explore all possible combinations of factor levels.

The experiments involved 3D printing runs for each combination of extrusion temperature and fan speed, with subsequent measurement and recording of tensile properties, alongside monitoring energy consumption for each run. This meticulous data collection aimed to establish a robust understanding of the relationships between the chosen parameters and the desired outcomes.

The subsequent statistical analysis, employing tools like Analysis of Variance (ANOVA), facilitated the identification of significant factors. This step was crucial in discerning which variables, among extrusion temperature and fan speed, significantly influenced tensile properties and energy consumption.

Upon identifying these influential factors, optimization techniques, such as desirability analysis, were applied to determine the optimal combination of extrusion temperature and fan speed that would yield the maximum tensile properties while concurrently minimizing energy consumption.

Each input parameter had three levels, namely printing temperature (170 °C/180 °C/190 °C) and fan speed (60%/80%/100%), resulting in a total number of 9 groups of experiments (Table 3).

To enhance sustainability in manufacturing, a decision-making method is essential, capable of concurrently addressing requirements related to energy consumption and mechanical properties. In this study, the desirability approach is applied to optimize printing parameters (temperature and fan speed), ensuring a simultaneous consideration of objectives related to both energy efficiency and mechanical performance.

Desirability analysis provides values within a range of zero to one, with one indicating the highest level of suitability.

For the case when the importance is the same for each response, the composite desirability *D* is calculated with the formula [29]

(5) $$D={\left(d_{1}\cdot d_{2}\cdot \ldots .\cdot d_{n}\right)}^{1/n}$$

where *n* is the number of responses, *d_i_* represents the desirability for each individual response, calculated (for the case when the goal is to maximize the response desirability) as [29]

(6) $$\begin{array}{l} d_{i}=0,\;if\;y_{i}<L_{i}\\ d_{i}=\frac{\left(y_{i}-L_{i}\right)\cdot r_{i}}{\left(T_{i}-L_{i}\right)},\;if\;L_{i}\leq y_{i}\leq T_{i}\\ d_{i}=1,\;if\;y_{i}>T_{i} \end{array}$$

When the objective is to minimize the response, the formulas used are [4] as follows:

(7) $$\begin{array}{l} d_{i}=0,\;\mathrm{if}\;y_{i}>U_{i}\\ d_{i}=\frac{\left(U_{i}-y_{i}\right)\cdot r_{i}}{\left(T_{i}-L_{i}\right)},\;\mathrm{if}\;T_{i}\leq y_{i}\leq U_{i}\\ d_{i}=1,\;\mathrm{if}\;y_{i}<T_{i} \end{array}$$

*y_i_*, *T_i_*, *L_i_*, and *U_i_* represent the predicted value, target value, lowest value, and upper value, respectively, of the analyzed response.

## 3. Results and Discussion

### 3.1. Thermal Characteristics of Samples Evaluated by DSC Analysis

**Figure 7:**
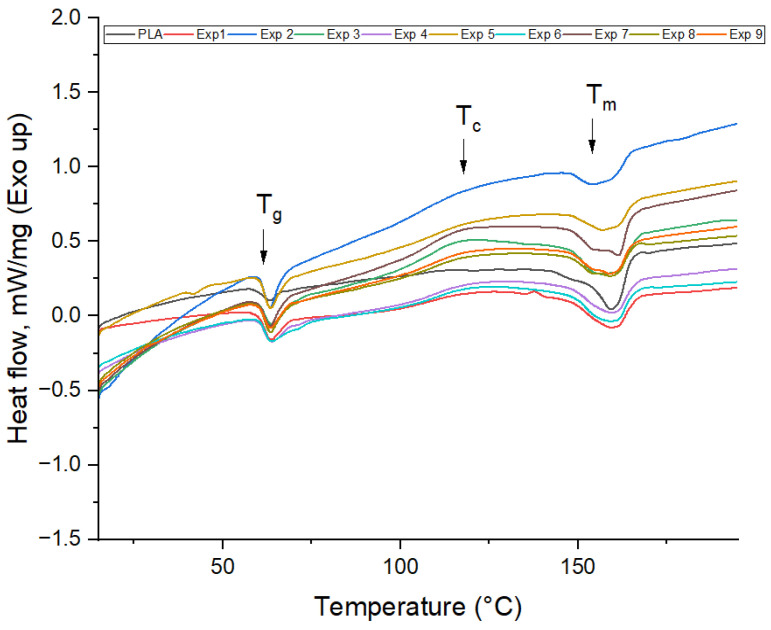
DSC curves for the 9 experiments (first heating scan).

The thermal characteristics of samples evaluated by DSC analysis are presented in Figure 7 and Table 4. All the samples exhibited three main phase transitions, which corresponds to the material glass transition temperature (*T_g_*), cold crystallization temperature (*T_c_*), and melting point (*T_m_*). As can be seen, regardless of the printing temperature and cooling rate, all experiments presented an endotherm glass transition peak (*T_g_*) around 63 °C, similar to the filament sample.

The cold crystallization process indicates the crystallization of a material during heating and is a typical behavior for aliphatic polyesters such as PLA, which, due to the structures of the macromolecules, does not crystallize easily [30]. The broad exothermic peaks associated with the cold crystallization temperature (*T_c_*) are observed within the temperature range of 118–124 °C. At a speed fan of 60%, the cold crystallization temperature *Tc* shifts to lower temperatures from 124.89 to 118.99 °C as the printing temperature increases from 170 to 190 °C due to increased mobility of molecular chains throughout the heating cycle [13]. Moreover, experiment no. 1 exhibited a second small exotherm crystallization peak at 140.66 ± 0.12 °C, which means that the low printing temperature of 170 °C and the low-speed fan of 60% caused the incomplete melting of PLA, so unmelted crystals were remelted and recrystallized during the DSC analysis [31]. A further increase in fan speed leads to similar behavior for experiments 4–9, namely the crystallization temperature increases with the printing temperature from 160 to 180 °C, followed by a decrease in *Tc* at the printing temperature of 190 °C. This fact can probably be due to insufficient time for the macromolecular chains to reorganize and form crystals at the temperature of 190 °C [32]. It should be mentioned that, regardless of the fan speed, the lowest values of the *Tc* temperature were recorded at a temperature of 190 °C.

Compared with the filament sample, which presents one sharp melting peak (*T_m_*) at 160.34 ± 0.22 °C, all the printed samples exhibit a broad peak with a maximum of 160 °C. In addition, for experiment nos. 7, 8, and 9, two melting peaks around 154 °C and 160 °C were observed. According to Y. Xu et al. [31] and B. Ma et al. [33], the double melting peak is a typical behavior for PLA, where the weaker and irregular crystals melt first and change into α crystals, after which the formed α crystals melt, resulting in the second *T_m_* peak. These results are consistent with data obtained from X-ray diffraction analysis, as seen in Table 4.

### 3.2. XRD Analysis

**Figure 8:**
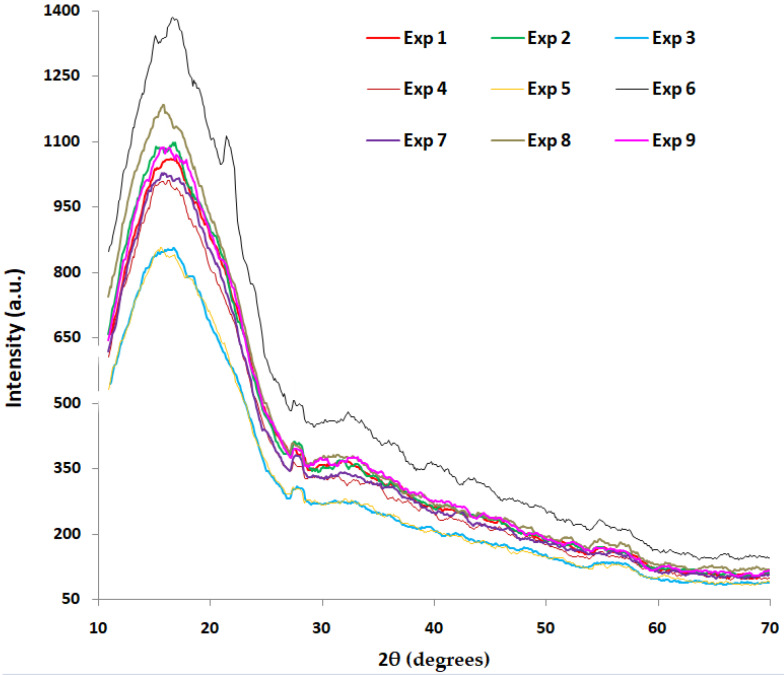
XRD patterns of 3D-printed samples.

Figure 8 shows the XRD patterns of 3D-printed samples. X-ray diffraction investigations show a change in the degree of crystallinity depending both on temperature and the rate of cooling (Figure 8). Semi-crystalline nature of colored thermoplastic aliphatic polyester is revealed by a broad peak (like a hump effect) in the range of 10–25° 2θ degrees.

The main reflections/peaks observed in the XRD spectra (Figure 8) around 2θ values of 14.79 (010), 16.62 (200/110), 19.03 (203/111), 27.34 (216), 29.43, 31.61, 41.12, correspond to α-phase of PLA belonging to orthorhombic system, according to the ICDD 00-064-1624 database. Other small peaks in the 2θ positions: 12.39 (004/103), 20.28 (204), 21.24 (015), 22.3 (015), 23.9 (016), and 24.95 (206), also confirm the PLA structure.

Based on the experimental data and adequate structural model, the lattice parameters were refined using the Rietveld method and Topas 4.1 program. From many crystal structures of the four polymorphs (α, α’, β, and γ) of colored thermoplastic aliphatic polyester solved over time starting from 1968 with the study of De Santis and Kovacs [34], it was assumed that an orthorhombic lattice similar to that reported by Aleman et al. [35] was obtained in our experiments. The lattice parameters *a*, *b*, and *c*, refined for the studied samples, were in the range *a* = 10.47–10.75 Å; *b* = 6.41–6.59 Å; and *c* = 27.50–28.14 Å (see Table 5), in good agreement with the literature [28,35,36,37,38]. The basic configuration of the colored thermoplastic aliphatic polyester crystal (α-phase) structure consists of two 10_3_ helices packed in an orthorhombic unit cell.

From Table 4, it can be observed that the values of crystallinity calculated from XRD were always higher than those calculated from DSC, similar to what was shown in a scientific paper [39].

The significant difference between the values of the crystallinity degree resulting from DSC and XRD, respectively, can be explained by the fact that the XRD technique scanned the sample surface, while the DSC technique evaluated the bulk crystallinity. The extrusion of PLA material in the printing process has modeled the morphology of the surface by flattening it and, as a result, the orientation led to higher crystallinity.

The values of the crystallinity degree calculated from the X-ray spectra show that the degree of crystallinity has a tendency to decrease with the rise of temperature and with the rise of the cooling rate (with the exception of 190 °C—80% sample, which shows the highest degree of crystallinity). It looks like lower printing temperatures associated with medium-lower cooling rates provide a higher degree of crystallinity. At higher printing temperatures, it must increase the cooling rate to maximize the degree of crystallinity.

### 3.3. Process Parameters Effect on Tensile Properties, Crystallinity and Energy Consumption

**Figure 9:**
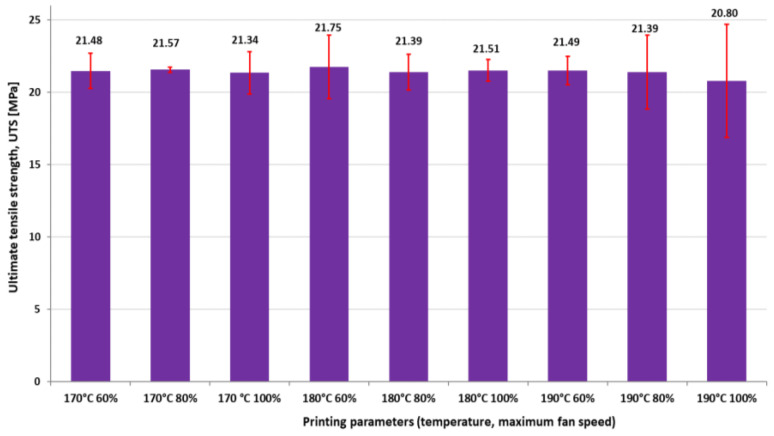
The influence of printing parameters on ultimate tensile strength of 3D-printed specimens.

**Figure 10:**
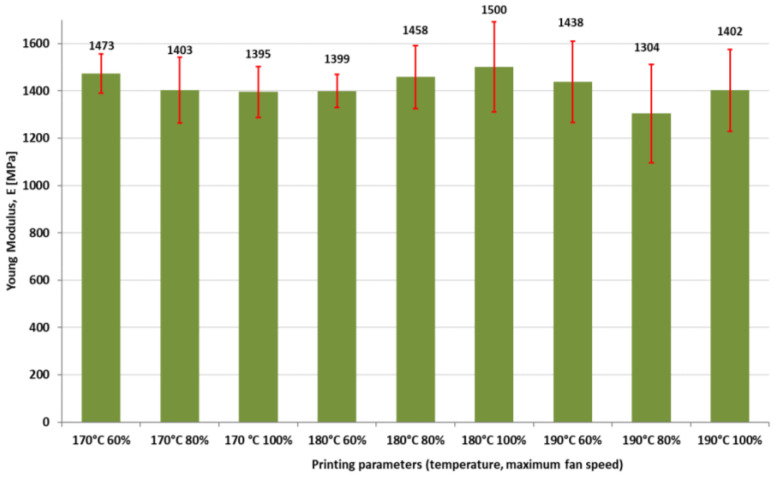
The influence of printing parameters on Young’s modulus of 3D-printed specimens.

**Figure 11:**
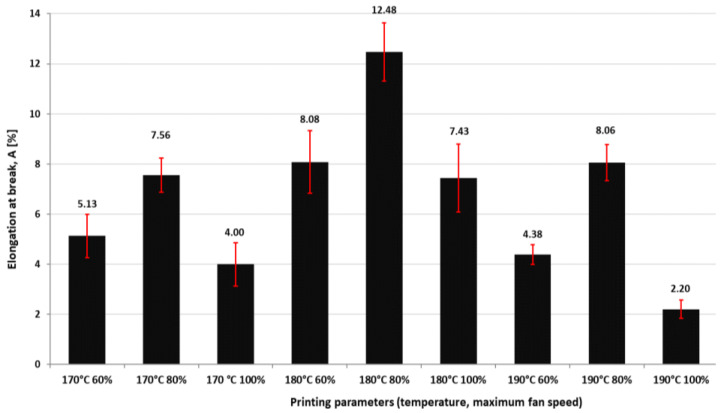
The influence of printing parameters on elongation at break of 3D-printed specimens.

**Figure 12:**
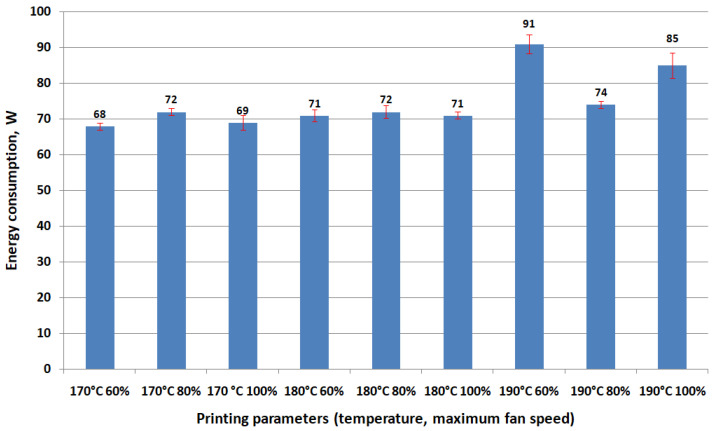
The influence of printing parameters on the energy consumed for 3D printing of specimens.

**Figure 13:**
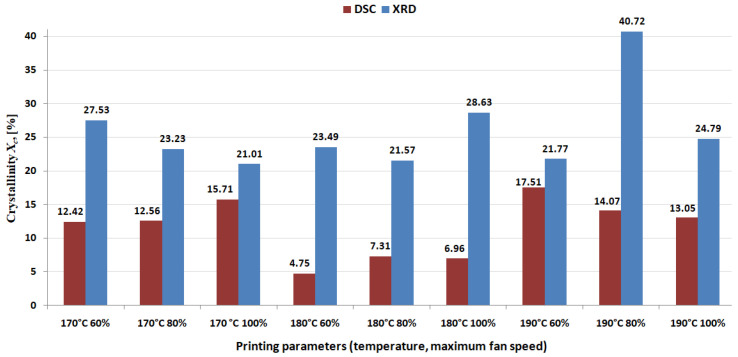
The influence of printing parameters on the crystallinity of 3D-printed specimens.

Figure 9, Figure 10 and Figure 11 present the results regarding the influence of printing parameters on the mechanical characteristics of 3D-printed samples, while Figure 12 and Figure 13 show the values of consumed energy and degree of crystallinity, respectively.

To establish a correlation between tensile properties and crystallinity of material, we need to examine how the mechanical properties of the material (such as ultimate tensile strength, Young’s modulus, and elongation at break) vary with changes in its crystallinity.

Based on the charts provided, the following observations can be made: generally, there seems to be a trend where materials with higher crystallinity exhibit greater tensile strength. For instance, at temperatures and fan speeds that lead to higher crystallinity (e.g., 190 °C and 60% fan speed), the tensile strength is also higher.

Young’s modulus appears to have a similar correlation with crystallinity. More crystalline materials generally have a higher Young’s modulus, indicating greater stiffness.

The elongation at break shows an inverse trend, materials with higher crystallinity seem to have lower elongation at break, suggesting that they are less ductile and more brittle.

About the crystallinity (XRD and DSC), there is a clear trend where increased temperature and decreased fan speed lead to higher crystallinity, measured by both X-ray diffraction (XRD) and differential scanning calorimetry (DSC).

To make a direct correlation, we should compare each set of printing parameters to the respective crystallinity values. For example, we could compare the tensile strength at different degrees of crystallinity to see if there is a linear or non-linear relationship between them. Similarly, we can compare Young’s modulus and elongation at break to crystallinity to better understand the relationship between the material structure and its mechanical behavior.

These correlations are crucial for understanding how the mechanical properties of a polymer change with its crystalline structure, which can be influenced by processing conditions such as temperature and cooling. Such understanding is vital for optimizing manufacturing processes and designing materials with specific mechanical properties required for particular applications.

The results suggest that, within the tested range of fan speeds and temperatures, 3D-printed samples exhibited relatively stable mechanical properties (UTS, A%, and E) with minor variations, very similar to samples printed with the provider’s recommended temperature of 210 °C as in previous authors studies [40].

Generally, the highest values of tensile modulus are obtained at 180 °C, while the lowest corresponds to 190 °C, and the highest value of Young’s modulus is obtained at 180 °C temperature and 100% fan speed.

Energy consumption increases with higher fan speeds and temperatures, which is important to consider in terms of cost and energy efficiency.

The obtained results regarding the crystallinity revealed that the printing temperature significantly influences this parameter, the highest values were obtained at 190 °C while the lowest values correspond to 180 °C. It can be observed that at 190 °C it was obtained, the lowest elongation at break, resulting in higher crystallinity, tends to reduce the flexibility. On the other hand, at 180 °C, the elongation at the break had the highest values; this result supports the statement presented before regarding the correlation between crystallinity and elongation at break. Complementary to this investigation, the authors conducted additional research into the tensile characteristics of specimens fabricated using a similar grade of polymeric-colored thermoplastic aliphatic polyester. Cross-referencing with findings from study [40], employing identical parameters of infill density (50%) and layer thickness (0.2 mm), but at a printing temperature of 200 °C, it can be observed marginal discrepancies, specifically a 9.81% difference in ultimate tensile strength (UTS) as compared to the UTS at 170 °C; a 9.43% difference compared to 180 °C; and a 10.8% difference relative to 190 °C extrusion temperature. These variations suggest that a reduction in extrusion temperature marginally influences the tensile strength of the printed samples. Conversely, reference [41] indicates that, under analogous conditions of a 0.2 mm layer thickness and a 200 °C extrusion temperature, the material exhibits an approximately 20% enhancement in Modulus of Elasticity (E) in comparison to median values reported in the present study, which may be attributed to divergent factors during printing, but still approximately in the same range. Additionally, in the same reference [2], the UTS showcases an approximate 15% decrement at an extrusion temperature of 200 °C relative to the average UTS measurements documented herein; this implies that reducing the temperature to improve energy efficiency can be successfully applied without compromising the tensile performance of the material.

### 3.4. Statistical Analysis

#### 3.4.1. Single Response Analysis

**Figure 14:**
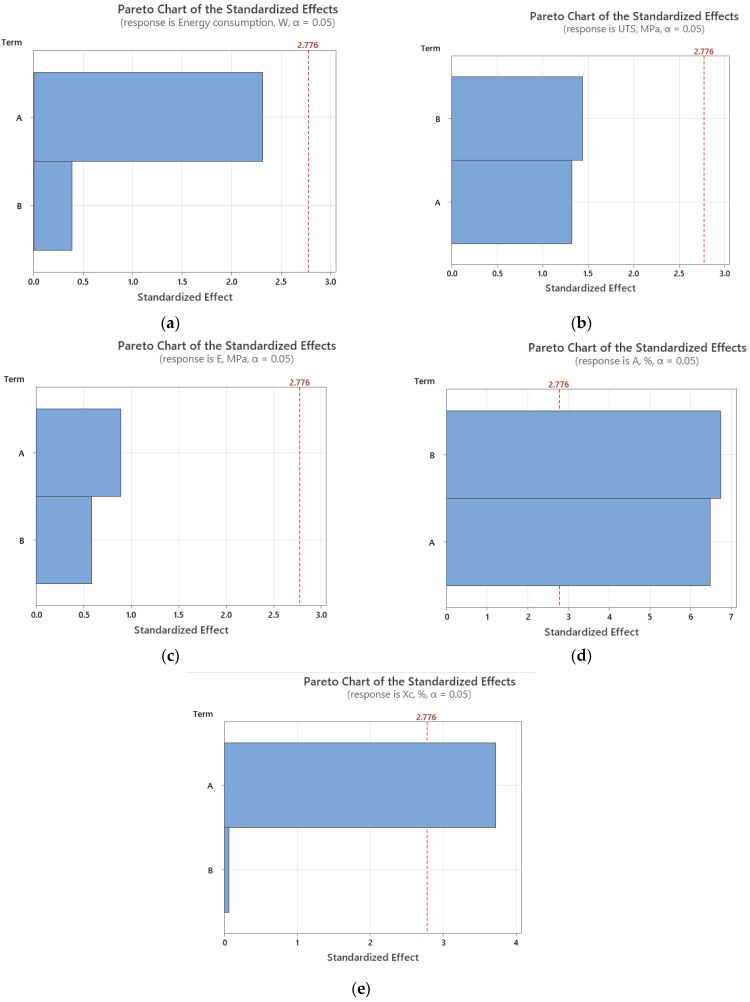
Pareto Charts for (**a**) energy consumption; (**b**) ultimate tensile strength; (**c**) Young’s modulus; (**d**) elongation at break; (**e**) crystallinity (terms: A: temperature, B: fan speed).

**Figure 15:**
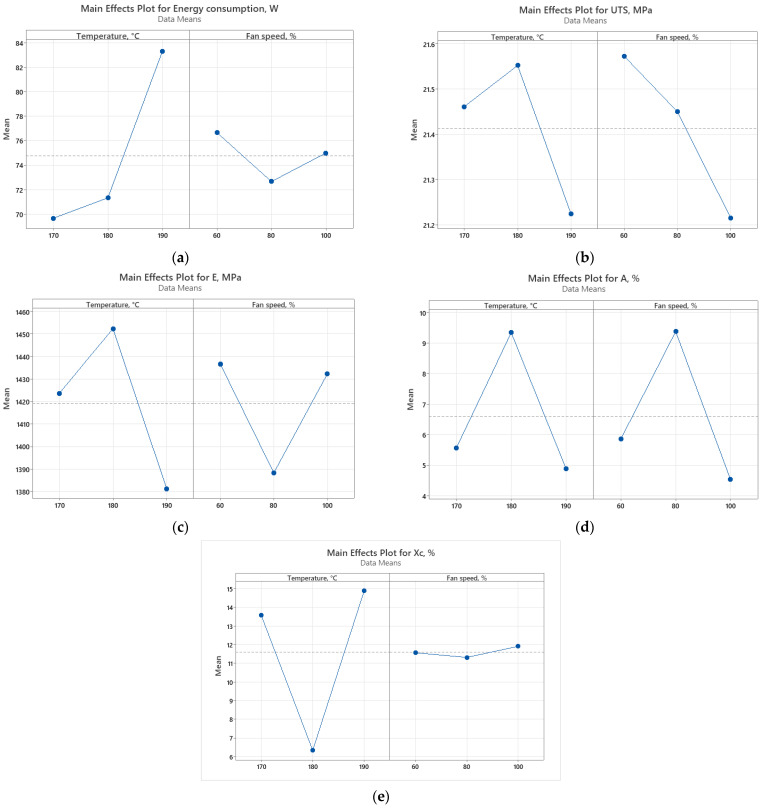
Main effect plots for (**a**) energy consumption; (**b**) ultimate tensile strength; (**c**) Young’s modulus; (**d**) elongation at break; (**e**) crystallinity.

To assess the impact of printing parameters (temperature and layer fan speed) on energy consumption and mechanical properties, Pareto charts (Figure 14) and main effect plots (Figure 15) graphical representations are illustrated.

It can be seen that the main factor that influences the energy consumption and tensile modulus is the temperature, while UTS and elongation at break are influenced both by temperature and fan speed, almost in the same way. Regarding the crystallinity, the printing temperature has a great influence, as also was concluded in [25], and the main effects plot for the degree of crystallinity is similar to the graph presented in [25], indicating that at intermediate printing temperature, the lowest value of crystallinity is obtained.

#### 3.4.2. Multi-Response Optimization

Optimization analysis was carried out to find the best combination of printing parameters to achieve maximum mechanical performance considering mechanical properties (*UTS*, *A*, *E*) and minimum energy consumption, as illustrated in Table 6.

Table 7 shows the ranks allocated to different options related to the printing parameters.

In Table 7, we observe that rank 1 corresponds to the experimental condition of 180 °C temperature and 80% fan speed, while rank 2 corresponds to the same temperature (180 °C) but with a slightly lower fan speed of 60%. The difference between the corresponding desirabilities is only 0.95%. The observation of similar composite desirability scores between these two conditions may initially seem unexpected, especially considering the Pareto chart’s indication that temperature is the most significant factor affecting energy consumption, tensile modulus, and crystallinity, while fan speed’s impact is less pronounced. These differences, although minor, are captured by the composite desirability analysis and reflected in the ranks assigned to each condition. Therefore, despite temperature being the dominant factor according to the Pareto chart, the combined effect of temperature and fan speed still warrants consideration in assessing the overall desirability of each experimental condition. The close ranks between conditions with similar temperatures but different fan speeds highlight the importance of comprehensively evaluating all factors to optimize the desired outcomes effectively.

**Figure 16:**
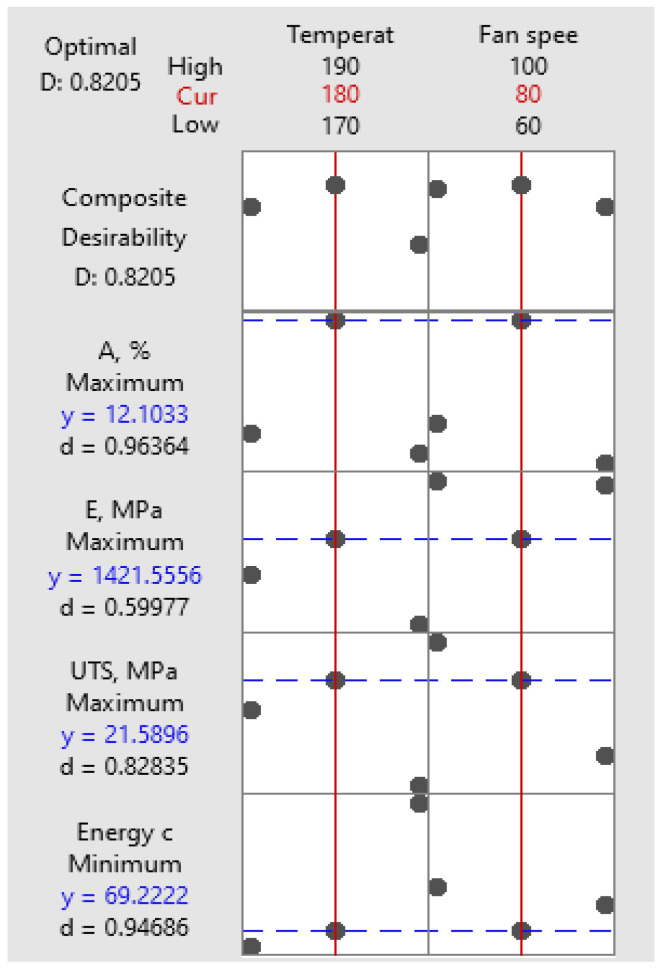
Optimization plot.

The optimization plot depicted in Figure 16 presents the impact of each factor (columns) on the responses or composite desirability (rows). Vertical red lines on the graph mark the current factor settings, and the numbers in red at the top of each column indicate the present factor level settings. The horizontal blue lines, along with accompanying numbers, signify the responses associated with the current factor level.

The optimization process yielded specific printing parameter values, namely 180 °C printing temperature and 80% fan speed, which are visually depicted in red on the optimization plot in Figure 16.

## 4. Conclusions

Based on the provided results, several general observations and conclusions can be made regarding the tensile properties of colored thermoplastic aliphatic polyester 3D-printed samples under different printing conditions:

The highest tensile modulus value was achieved at 180 °C temperature and 100% fan speed. This indicates that this specific combination of temperature and fan speed is favorable for obtaining the maximum stiffness or tensile modulus in 3D-printed products. Generally, higher values of tensile modulus were consistently obtained at 180 °C compared to other temperature settings. In contrast, smaller tensile modulus values were observed at 190 °C. This suggests that a temperature of 180 °C is ideal for achieving higher stiffness properties in colored thermoplastic aliphatic polyester prints, while temperatures exceeding this level may lead to reduced stiffness.

The highest elongation at break value was obtained at 180 °C temperature and 80% fan speed. This combination allowed the analyzed material to exhibit the greatest deformation or ductility before breaking. Similar to the findings for tensile modulus, higher values for elongation at break were generally associated with a temperature of 180 °C, while smaller values were observed at 190 °C. Additionally, it was noted that the elongation at break values at 190 °C was nearly equivalent to those obtained at 170 °C. This indicates that temperatures above 180 °C may not provide significant benefits in terms of elongation at break.

The ultimate tensile strength remained relatively similar for all tested printing conditions. This suggests that the UTS of PLA is relatively consistent across the range of parameters investigated. The parameters that exhibited notable changes were the elastic modulus and elongation at break.

Temperature significantly impacts energy consumption and tensile modulus, while fan speed is a key factor for UTS and elongation at break. On the other hand, the degree of crystallinity of 3D-printed specimens is highly influenced by the printing temperature. The examination of crystallinity via both DSC and XRD analyses reveals intricate relationships influenced by temperature and fan speed. The substantial differences observed between DSC and XRD crystallinity values underscore the distinct sensitivities of these techniques to various aspects of the material.

Furthermore, the initial hypothesis of the study was confirmed: lower printing temperature than those recommended and different cooling fun capacities result in the similar mechanical performance of obtained samples and simultaneously determine optimized energy consumption. Further studies will involve other methods to obtain energy efficiency for the 3D printing process as well as the decrease in material consumption when obtaining 3D-printed products leads to both energy savings and a positive impact on the environment.

In summary, the optimal conditions for maximizing tensile modulus and elongation at break in colored thermoplastic aliphatic polyester 3D-printed samples appear to involve a temperature of 180 °C. While ultimate tensile strength remains consistent across various conditions, the choice of temperature and fan speed has a significant impact on the elastic modulus (stiffness) and elongation at break (ductility) of the printed material. These conclusions can be valuable for selecting appropriate printing parameters based on specific mechanical property requirements for PLA-based 3D-printed components.

When we delve into the specifics of factors like extrusion temperature and fan speed in 3D printing, we uncover their direct implications on energy consumption and environmental impact. Adjusting the extrusion temperature can have a significant effect on the energy required to melt and deposit the filament. Higher temperatures generally result in higher energy consumption due to increased heat transfer requirements. By fine-tuning the temperature to the optimal level for the specific material being used, we can reduce energy waste and improve overall printing efficiency. Fan speed plays a crucial role in the cooling process of the printed layers. Higher fan speeds generally consume more energy but help with faster cooling and better print quality. However, by optimizing the fan speed based on the complexity of the print and the material being used, we can strike a balance between energy efficiency and print quality.

In the broader context of the green transition, these seemingly minor adjustments can accumulate into substantial energy savings across the 3D printing industry. By conducting thorough studies and experiments to understand the energy implications of these factors, manufacturers and users can not only optimize their printing processes but also contribute to sustainable practices that align with environmental goals and initiatives. By providing a comprehensive analysis of the interplay between extrusion temperature, fan speed, tensile properties, and energy consumption, coupled with the application of DOE to systematically clarify their intricate relationships, the present work contributes a unique dimension to the understanding of 3D printing processes and aligns with the broader goal of achieving more sustainable and efficient additive manufacturing practices.

## Figures and Tables

**Table 1:** Characteristics of colored thermoplastic aliphatic polyester filaments from providers’ data sheets.

Extrusion Temperature (°C)	Bed Temperature (°C)	Density (g/cm^3^)	Tensile Strength (MPa)	Specific Deformation (%)	Charpy Impact Strength (kJ/m^2^)
210 ± 10	25–60	1.31 ± 0.02	15.5–72	34.5 ± 8.1	5.7 ± 0.4

**Table 2:** Printing parameters used for specimen fabrication.

Shell Width (mm)	1
Infill percentage (%)	50
Infill speed (mm/s)	70
Estimated print time (min)	46
Estimated filament used (g)	10.6
Extruder temperature	160 °C, 170 °C, 180 °C
Bed temperature (°C)	60
Platform addition	Raft only

**Table 3:** DOE array.

Experiment No.	Fan Speed, %	Printing Temperature, °C
1	60	170
2		180
3		190
4	80	170
5		180
6		190
7	100	170
8		180
9		190

**Table 4:** Thermal characteristics of the analyzed samples.

Experiment No.	*T_g_*, °C	*T_c_*, °C	*T_m_*, °C	*X_c_*, %	
				DSC	XRD
1	63.45 ± 0.25	124.89 ± 0.15	160.30 ± 0.5	12.42	27.53
2	63.17 ± 0.34	122.74 ± 0.17	160.67 ± 0.25	4.75	23.49
3	63.82 ± 0.15	118.99 ± 0.18	160.57 ± 0.15	17.51	21.77
4	63.95 ± 0.15; 69.81 ± 0.23	122.74 ± 0.21	160.17 ± 0.32	12.56	23.23
5	63.51 ± 0.36	124.32 ± 0.15	160.83 ± 0.27	7.31	21.57
6	63.46 ± 0.27; 70.5 ± 0.32	120.56 ± 0.11	160.32 ± 0.25	14.07	40.72
7	63.48 ± 0.47	119.16 ± 0.28	154.5 ± 0.13; 160.63 ± 0.27	15.71	21.01
8	63.48 ± 0.28	121.25 ± 0.18	160.40 ± 0.18; 154.3 ± 0.12	6.96	28.63
9	63.15 ± 0.31	118.17 ± 0.13	160.41 ± 0.12 154.5 ± 0.19	13.05	24.79

**Table 5:** Lattice parameters of α-phase of investigated sample.

Sample	*a* (Å)	*b* (Å)	*c* (Å)	Volume (Å^3^)
170 °C—60%	10.69	6.47	27.95	1933.14
180 °C—60%	10.56	6.58	28.05	1949.05
190 °C—60%	10.69	6.48	27.70	1918.81
170 °C—80%	10.75	6.52	27.50	1927.47
180 °C—80%	10.47	6.59	27.79	1917.43
190 °C—80%	10.72	6.41	28.14	1933.64
170 °C—100%	10.71	6.43	28.05	1931.67
180 °C—100%	10.66	6.52	27.86	1936.36
190 °C—100%	10.67	6.50	27.82	1929.45

**Table 6:** Optimization goals for analyzed characteristics.

Response	Goal	Lower	Target	Upper	Weight	Importance
*A*, %	Maximum	2.20	12.48		0.25	1
*E*, MPa	Maximum	1304.00	1500.00		0.25	1
*UTS*, MPa	Maximum	20.80	21.75		0.25	1
*E_c_*, W	Minimum		68.00	91	0.25	1

**Table 7:** Composite desirability and ranks.

Temperature, °C	Fan Speed, %	Composite Desirability	Rank
170	60	0.880549084	5
170	80	0.903883896	4
170	100	0.816274952	6
180	60	0.942813925	2
180	80	0.951754815	1 *
180	100	0.904625334	3
190	60	0.766475233	8
190	80	0.796147753	7
190	100	0.682569715	9

* The highlighted line corresponds to the optimal values (having Rank 1) of printing settings.

## Data Availability

Data are contained within the article.

## Supplementary Materials

The following supporting information can be downloaded at: https://www.mdpi.com/article/10.3390/polym16101364/s1, Figure S1: TGA curve for filament.
