# Supplementary figures and images for: Analyzing Sustainable 3D Printing Processes: Mechanical, Thermal, and Crystallographic Insights
Source: Polymers (Basel). 2024 May 10;16(10):1364. doi: 10.3390/polym16101364 (PMC11125246; doi:10.3390/polym16101364)

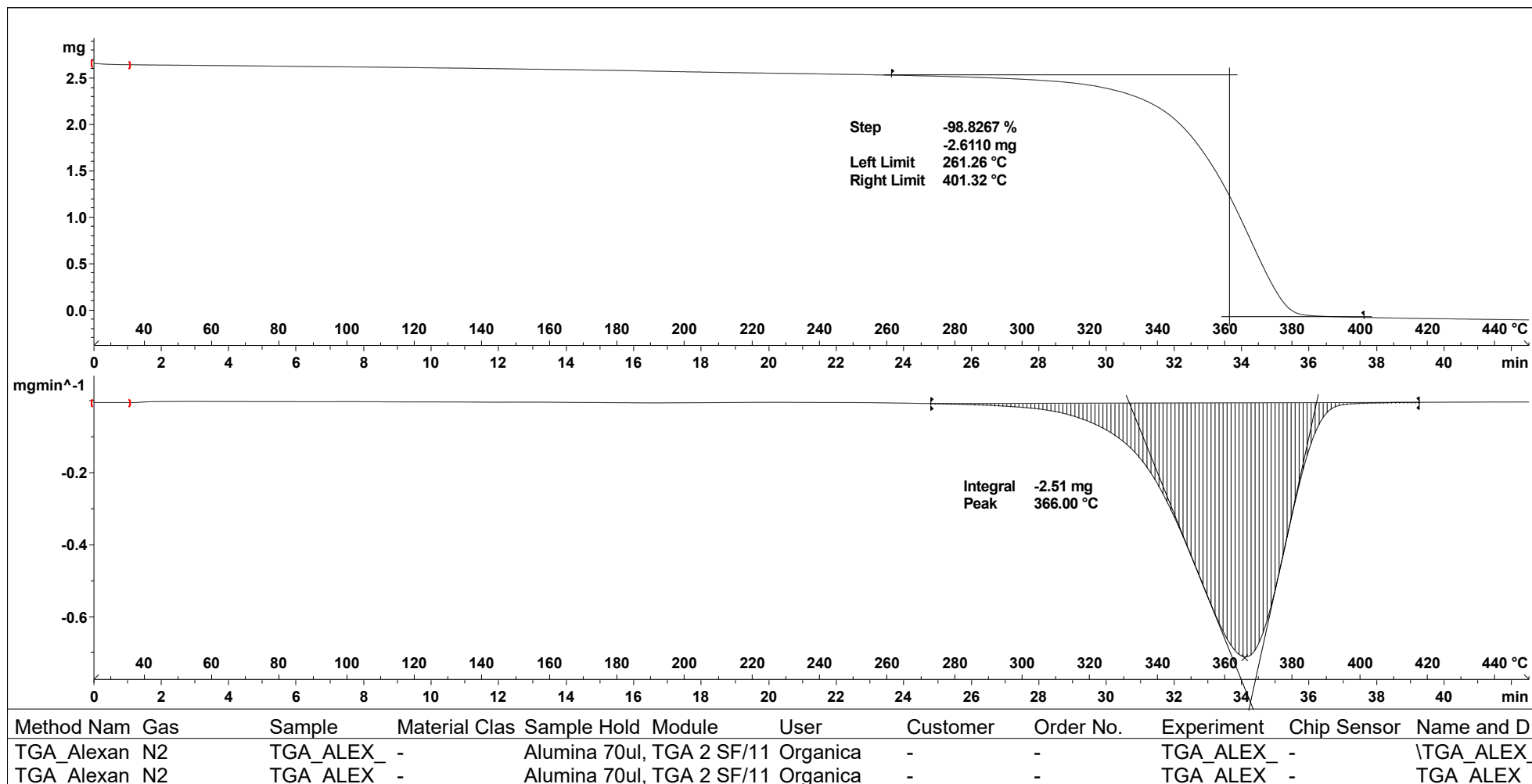

Lab: Organica

STAR® SW 17.00

Supplement: Supplementary file 1 [file polymers-16-01364-s001.zip › polymers-2906738-supplementary.pdf]
